# Supplementary material for: Single-Cell and Bulk RNA Sequencing Reveal SPINK1 and TIMP1 as Epithelial Cell Marker Genes Linked to Colorectal Cancer Survival and Tumor Immune Microenvironment Profiles
Source: Int J Mol Sci. 2025 Dec 11;26(24):11964. doi: 10.3390/ijms262411964 (PMC12733225; doi:10.3390/ijms262411964)
Supplement: Supplementary file 1 [file ijms-26-11964-s001.zip › Supplementary Figure.pdf]

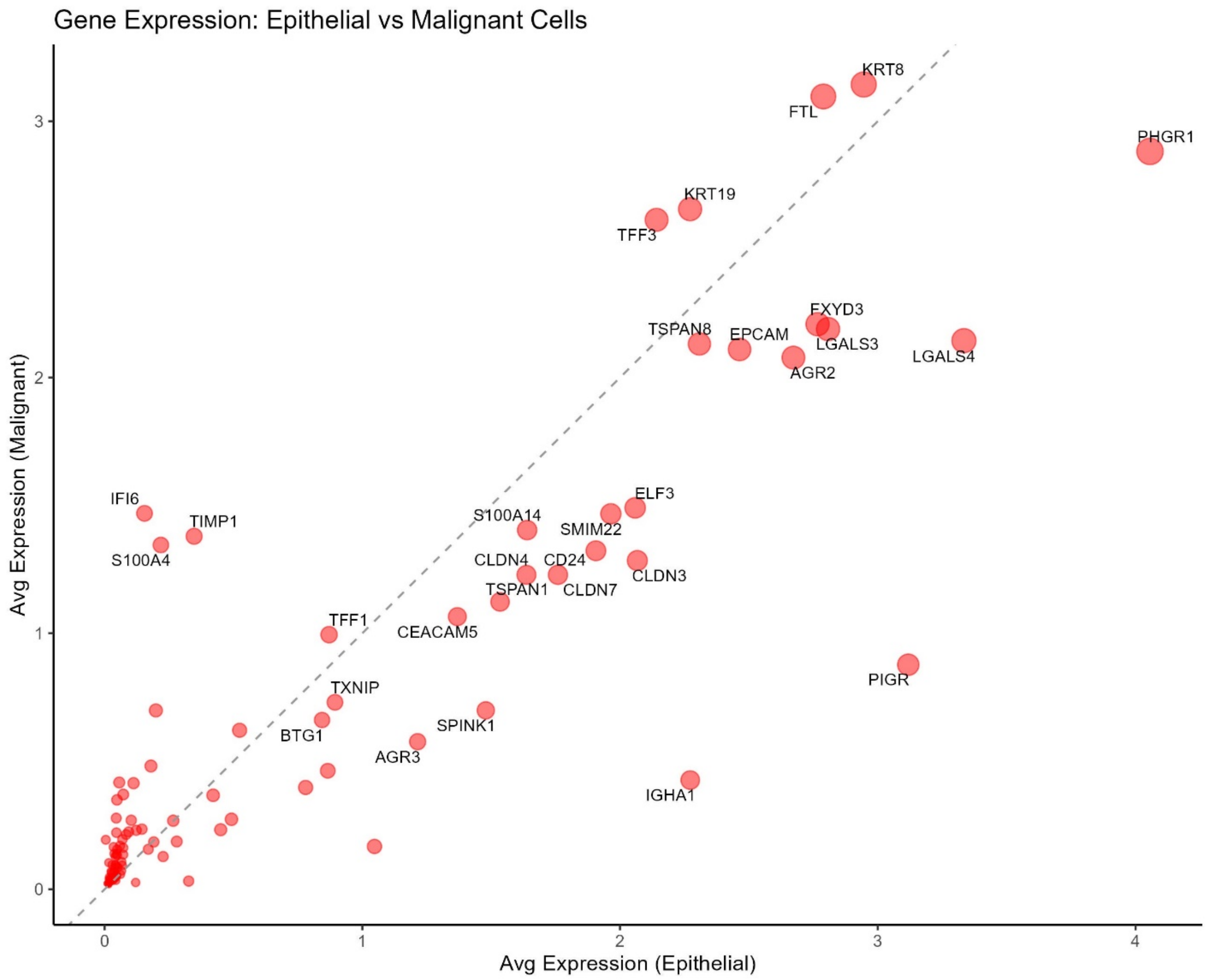

Figure S1. Scatter plot showing the overlapped genes between the epithelial cell cluster and the malignant cell cluster.
